# Retraction: A comparative randomized clinical trial evaluating the efficacy and safety of tacrolimus versus hydrocortisone as a topical treatment of atopic dermatitis in children

**DOI:** 10.3389/fphar.2026.2021934

**Published:** 2026-10-14

**Authors:** Frontiers Editorial Office

The journal retracts the 20 September 2023 article cited above.

Following publication, concerns were raised regarding data misrepresentation.

Following provision of raw data by the authors, the Chief Editor concluded that the article’s conclusions and assertions were not sufficiently supported by the findings from the material provided; therefore, the article has been retracted.

This retraction was approved by the Chief Editors of Frontiers in Pharmacology and the Chief Executive Editor of Frontiers.

The authors do not agree with this retraction.

Frontiers would like to thank André Gillibert, Hywel C. Williams and Florian Naudet who contacted us regarding the published article.

